# Advantages of melodic over rhythmic movement sonification in bimanual motor skill learning

**DOI:** 10.1007/s00221-017-5047-8

**Published:** 2017-07-26

**Authors:** J. F. Dyer, P. Stapleton, M. W. M. Rodger

**Affiliations:** 1 School of Psychology, Queen’s University Belfast, Belfast, UK; 2 School of Arts, English and Languages, Queen’s University Belfast, Belfast, UK

**Keywords:** Bimanual coordination, Skill, Retention, Augmented feedback, Movement sonification

## Abstract

An important question for skill acquisition is whether and how augmented feedback can be designed to improve the learning of complex skills. Auditory information triggered by learners’ actions, movement sonification, can enhance learning of a complex bimanual coordination skill, specifically polyrhythmic bimanual shape tracing. However, it is not clear whether the coordination of polyrhythmic sequenced movements is enhanced by auditory-specified timing information alone or whether more complex sound mappings, such as melodic sonification, are necessary. Furthermore, while short-term retention of bimanual coordination performance has been shown with movement sonification training, longer term retention has yet to be demonstrated. In the present experiment, participants learned to trace a diamond shape with one hand while simultaneously tracing a triangle with the other to produce a sequenced 4:3 polyrhythmic timing pattern. Two groups of participants received real-time auditory feedback during training: melodic sonification (individual movements triggered a separate note of a melody) and rhythmic sonification (each movement triggered a percussive sound), while a third control group received no augmented feedback. Task acquisition and performance in immediate retention were superior in the melodic sonification group as compared to the rhythmic sonification and control group. In a 24-h retention phase, a decline in performance in the melodic sonification group was reversed by brief playback of the target pattern melody. These results show that melodic sonification of movement can provide advantages over augmented feedback which only provides timing information by better structuring the sequencing of timed actions, and also allow recovery of complex target patterns of movement after training. These findings have important implications for understanding the role of augmented perceptual information in skill learning, as well as its application to real-world training or rehabilitation scenarios.

## Introduction

Performance of learned motor skills requires the perception and use of task-relevant information via the senses. Generally speaking, availability of more precise perceptual information can enable finer control of movement and more effective task performance (Mechsner et al. 2001; Todorov et al. 1997; Wilson et al. 2005). This improvement in performance can be afforded by the provision of concurrently-presented augmented feedback, which tracks some property of movement (for example, deviation from a desired trajectory, e.g., Sigrist et al. 2013b), and presents this back to the mover in real time. The availability of higher quality information allows enhanced performance-monitoring, by making errors more salient and correctable. When used in a motor skill learning scenario, augmented feedback can lead to better learning outcomes than would be possible in the absence of feedback (Sigrist et al. 2013a). To acquire a perceptual-motor skill, the learner must attune his/her attention to the perceptual information which is most relevant for effective completion of the task at hand (Gibson 1969, 1988). Augmented perceptual information (as provided by concurrent feedback) may speed up this ‘education of attention’ by highlighting specifically the information which is relevant for the task, i.e., information for the control of the body or a tool. Although most research into the effectiveness of augmented feedback has used visual displays to provide information for task performance (Kovacs et al. 2009; Vander Linden et al. 1993), auditory displays have also been shown to be effective as concurrent augmented feedback (Dyer et al. 2016). ‘Movement sonification’, live sound controlled by learner movement, is a potentially useful approach to providing a learner with task-relevant augmented feedback to enhance the acquisition and retention of complex motor skills (Dyer et al. 2015; Effenberg 2005).

Previous experimental investigations of augmented feedback for complex motor skill learning showed that providing feedback can improve performance when it was available, but may lead to substantial performance decrements in subsequent retention testing in which augmented feedback is removed (Anderson et al. 2005; Maslovat et al. 2009). This finding has become known as the ‘guidance effect’, and is hypothesised to be the result of over-reliance on the augmented feedback at the expense of tuning into task-intrinsic information. However, more recent research which uses sonification to provide auditory information for task performance (rather than visual information) has not shown a guidance effect, questioning the universality of this effect for augmented feedback (Dyer et al. 2016; Ronsse et al. 2011; van Vugt and Tillmann 2015).

Avoidance of the guidance effect with movement sonification as augmented feedback for motor skill learning is unlikely due to the modality of presentation (visual versus auditory), but rather the informational structure by which movement patterns are presented to the learner. In the examples cited above, individual sounds are triggered by each completed movement of participants, signalling the overall temporal pattern of sequences of consecutive movements. Thus, there is a one-to-one mapping between the auditory form of the feedback and the actions of the learner. In typical visually-presented augmented feedback, when it is displayed as a live graph or a Lissajous display, a transformation of the information required to perform the task in the absence of feedback occurs (Kovacs et al. 2010), meaning that the skill which is learned is how to perceive and control the display, rather than the kinematics of limb movement. In effect, this means that performance in practice (with feedback) and retention (without feedback) is two different tasks and, as such, improved performance in one may not generalise to the other. In contrast, feedback which does not alter the dynamics of the perception–action task at hand produces performance increments which are resistant to the removal of feedback (Chiou and Chang 2016; Wilson et al. 2010). It follows then that effective augmented feedback should not transform the information which is necessary to perceive in a naturalistic performance scenario (i.e., without feedback). In fact, the primary role for feedback (if the goal is learning which is not dependent on feedback) should be to highlight relevant features of task-intrinsic sensory information, i.e., to technically be redundant. Vinken et al. (2013) stress that sonification as augmented feedback should share a strong temporal–structural correlation with intrinsic information sources to take advantage of multisensory integration processes.

Dyer et al. (2016) showed that non-abstracted, redundant sonification of movement enhances performance in a bimanual motor skill (4:3 rhythmic shape tracing—the same task used in the current experiment). This task involved tracing two regular shapes on a workspace with the index finger of both hands concurrently (a triangle for the left hand and a diamond for the right). Learners were required to make regular movements between shape corners so as to produce an inter-corner bimanual timing ratio of 4:3. In one experimental condition, fingertip arrivals at corner zones were sonified—each producing an enveloped burst of pure tone, mapped to a specific pitch. When performed correctly, one full sequence produced a simple melody. Prior to each practice trial, learners were shown a visual demo animation of correct performance, and sonified with the same mapping. Practice trials thereby became an attempted musical performance, as learners tried to match both the visual and auditory elements of the demo, and “play” the task correctly. As expected, when live sonified feedback was withdrawn, learners maintained the high level of performance that they had reached by the end of practice. Performance in the sonification condition was better than in a control condition, in which any audible effect of learners’ movements was masked by continuous pink noise throughout the practice phase (also during the demo presentation). Another condition, in which only the demo was sonified, and learner movement was not, did not improve learning any better than the control condition. This indicated that the benefit of sound in the task was due to the self-generated perceptual information that could be compared to the target pattern—rather than a perceptual unification of the activity of both hands into an easier-to-perceive musical gestalt (Franz et al. 2001). Learners were able to perform the task to the same practiced standard under naturalistic conditions, i.e., without feedback (however, this effect was only short term, disappearing after 24 h).

Although the results of Dyer et al. (2016) suggest enhancement of complex bimanual coordination skill learning using melodic movement sonification as feedback, improvement with sonification condition could simply be the result of self-produced relative timing information, irrespective of the melodic nature of the sonification mapping. Kennedy et al. (2013) have shown that simple beeps triggered by finger taps can enhance the learning of polyrhythmic bimanual tapping coordination. It is possible that the same benefit would be found in the more complex bimanual coordination task used by Dyer et al. (2016), i.e., if shape corner arrivals were sonified with identical bursts of sound, devoid of any corner-specific pitch content. This question is addressed by the current experiment, by comparing a melodic sonification condition with another sonification condition in which corner arrivals are sonified using short, percussive bursts of white noise. If this “rhythmic sonification” condition produces performance benefits relative to control, then it might suggest that action-coupled sound per se can be sufficient for performance enhancement in rhythmic, continuous tasks such as this. Alternatively, there could be a performance advantage only in the “melodic sonification” condition, which would indicate that the melody creates a more unified Gestalt by which the patterning of different individual movements that the shape tracing entails can be grouped.

The task-sonification mapping for this task suggests a benefit for melody over rhythm, based on information content. In this bimanual task, each corner of each shape is assigned a different tone. Played in order, they produce the melody heard in the demo presentation. This means that mistakes in the ordering of bimanual movements effectively stand out as incorrect. A movement out-of-order means a note out-of-order in the increasingly familiar melody, and this mistake can be corrected on the next cycle. In the proposed rhythmic sonification condition, the salience of ordering errors is unlikely to be as great. If learners use this melodic strategy to learn the correct ordering of hand movements, it should manifest as a faster rate of learning in the proposed melodic sonification condition than the rhythmic sonification condition. If so, this result could serve as evidence-based justification for the use of melody in sequential motor skill learning with sonification. Hence, it is hypothesised that there will be greater enhancement of learning for melodic feedback over rhythmic feedback.

A further concern in motor skill learning is the retention of good performance beyond the time period immediately following the practice phase. Delayed retention tests are essential to determine whether improved performance is a short-term effect, or can reasonably be called learning (Salmoni et al. 1984; Soderstrom and Bjork 2015). Although learners in the movement sonification group of Dyer et al. (2016) retained the goal movement pattern 5 min after training, their performance levels were statistically indistinguishable from control participants 24 h following practice. Many participants in the sonification condition reported being unable to remember the melody from the previous day’s practice, and claimed that this was the reason for their perceived poorer performance (no feedback was given). One solution which could remedy the 24-h drop-off in performance would be to allow learners to listen to a replay of the sound of perfect performance before a delayed retention test—a strategy which has been effective in piano training (Lahav et al. 2013).

There is reason to hypothesise that listening to the training melody may assist in delayed recovery of the target movement pattern. Neurally, fMRI activation crossover between perception of music and perception/performance of movement is substantial, especially for learned music (Bangert et al. 2006; Chen et al. 2012; Lahav et al. 2007; Lotze et al. 2003). This effect likely applies beyond the domain of learned music to action-relevant sound more generally (Cesari et al. 2014). The skilled listener, i.e., one who has learned the mapping between movement and sound in an interactive sonification environment, should be able to perceive useful information from a replay of the sound of perfect motor performance. For the current experiment, it is hypothesised that on a 24-h retention test, participants will show improved motor performance following a short listening period (pre-recorded sound only, no visual presentation or live movement sonification). This finding would show that sonification-enhanced training can be ‘refreshed’ by listening to the sound of good performance.

The aims of the present experiment are to investigate two separate questions of sonification for motor skill learning. The first is whether there is a specific benefit to using a musical, melodic sonification in ordered, sequential tasks such as the task to be learned here—as opposed to a sonification strategy which provides non-melodic, rhythmic information only. To address this, three experimental conditions are used. A “melodic sonification” condition, in which movement events are sonified using notes which together, and in order, form a melody, and a “rhythmic sonification” condition, in which the same movement events are sonified using identical bursts of white noise. These are compared to each other and a control condition, in which no auditory feedback of any kind is provided. To mask any residual self-produced sounds from the task, participants in this condition hear constant pink noise at a comfortable volume. The second question is the extension of good performance beyond an initial retention test on the day of practice. Participants in all three conditions are tested twice on day 2. The second such test is preceded by an auditory playback of the demonstration (melodic or rhythmic depending on the condition). The control condition will complete two identical retention tests on day 2, with no demo presentation of any kind. This is intended to address the issue of a potential practice effect due to multiple retention tests in a short time period. By comparing the change in performance on the control condition with the other two conditions (post-replay), it should be possible to reasonably assert whether the change was due to repeated performance, i.e., practice, or from the sonic replay.

## Methods

### Participants

An opportunity sample of 60 participants (39 female, 21 male) was recruited from a pool of undergraduate psychology students, post-graduate researchers and staff in the university at which the experiment was conducted. Undergraduate students received course credit for their participation where applicable. Only right-handed participants were recruited, as confirmed by administration of the Edinburgh Handedness Inventory (Oldfield 1971). Handedness scores did not differ significantly between the three experimental groups (*F*(2, 59) = 0.260, *p* = 0.772).

Participants were asked to report any musical experience or participation in dance activity. No professional musicians or experienced drummers were included in the sample. In the control condition, there were eight participants with some musical experience (mean 8 years, SD = 3.91), four of whom were currently in some way involved in music (e.g., recreational players/learners) and one participant who was a regular dancer. In the rhythmic sonification condition, there were eight participants with musical experience (mean 9 years, SD = 3.16), three of whom were currently involved in music, with one dancer. In the melodic sonification condition, there were nine participants with musical experience (mean 6.33 years, SD = 3.28), two of whom were currently involved in music.

Informed consent was obtained from all individual participants included in the study.

## Materials and apparatus

**Fig. 1:**
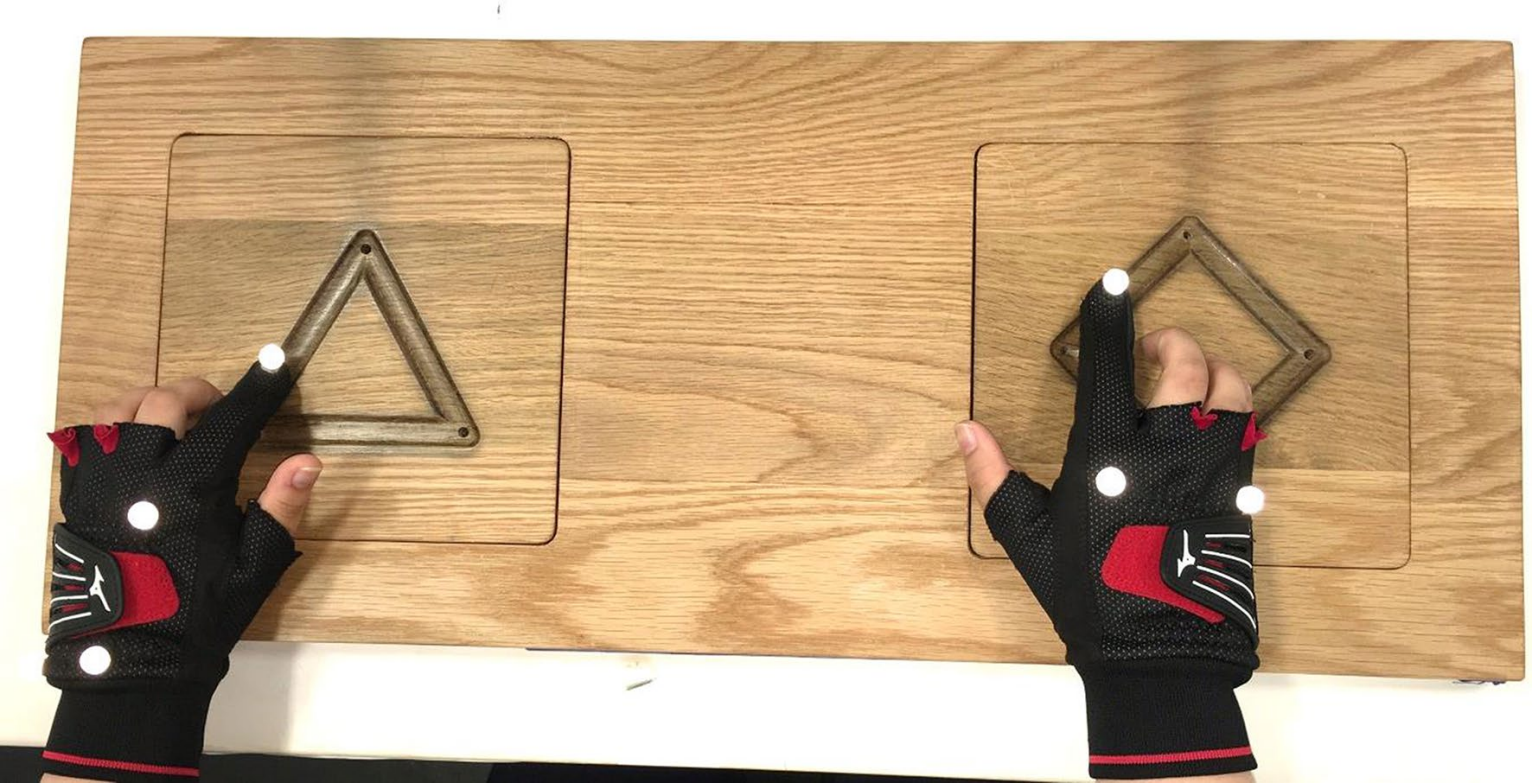
Board on which participants practiced the bimanual motor skill. Shapes were traced concurrently and movements were tracked by the position of reflective markers on the fingertips. Fingertip arrivals at corner zones were taken as the basis for performance measurement and sonification

The hardware used in this experiment is identical to that used in Dyer et al. (2016). A bespoke wooden board was used (Fig. 1), with two regular polygons cut into the top side. A triangle was positioned on the left and a square-diamond on the right. The centre points of these shapes were roughly shoulder-width apart to facilitate concurrent bimanual tracing with the index finger of both hands.

Participants interacted with the board while seated at a desk with the board positioned directly in front at a comfortable height. Visual features of the demonstration were presented on a 17-inch screen positioned approximately 2 m in front of participants. Auditory features were presented through a pair of Sennheiser headphones, which were worn at all times during the experiment. Participants additionally wore a pair of gloves with all fingers removed except the index finger (Fig. 1). Small reflective markers were attached to these gloves, so that they could be individually identified and the fingertip of each hand tracked in three dimensions.

Hand movement was captured with a set of four optical motion capture cameras (Qualisys) capturing at 300 Hz. This system provided the three-dimensional data for both live sonification and later movement analysis.

The experiment was administered from a desktop PC running Qualisys Track Manager. Three-dimensional Cartesian movement data were streamed via the OSC protocol to a Max/MSP 6.0 patch, which provided sonification based on movement of the index finger marker. A demo animation and terminal (post-trial) graphical feedback display were produced using Processing.

Three kinds of feedback were provided in the current experiment. Concurrent melodic sonification and rhythmic sonification were available to participants during practice trials in those experimental conditions. Terminal feedback in the form of a graph of performance was provided to all participants (including those in the control condition) following every trial. All three kinds of feedback are based on the same movement events: index fingertip arrivals at corner zones of the shapes.

**Fig. 2:**
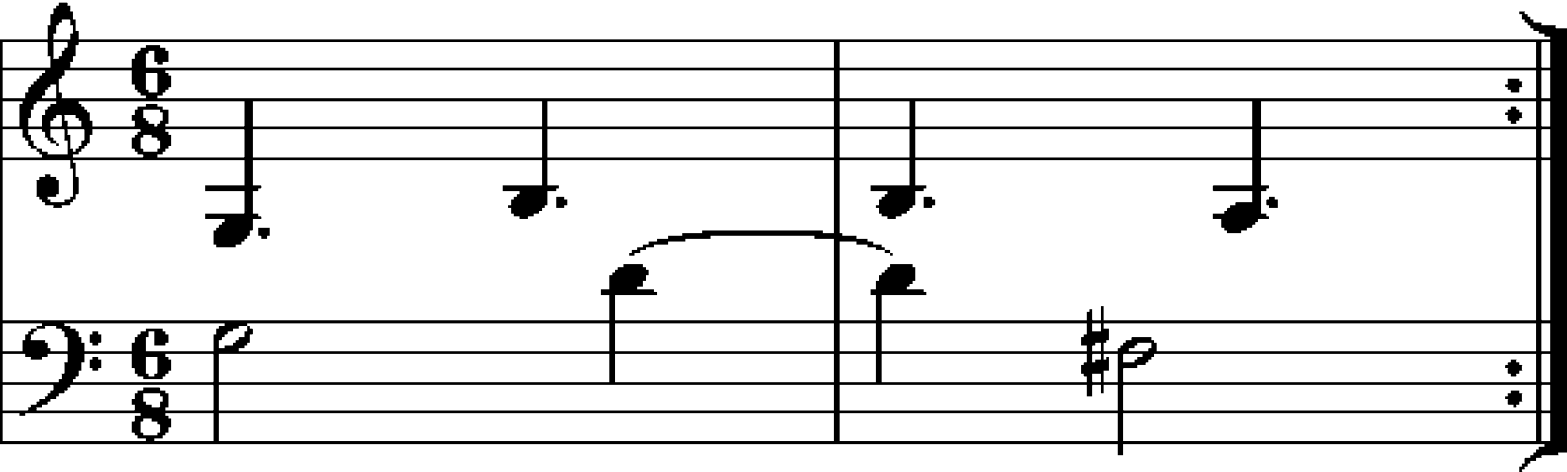
Demonstration melody presented in the melodic sonification condition and produced by correct performance by participants in the same condition. When movements were sonified, *right*-hand movements produced notes from the *upper row* and *left* from the *lower*

When the index finger of either hand entered a zone defined around a shape corner, a trigger signal was generated in Max/MSP. In the melodic sonification condition, this produced a note from the melody, as shown in Fig. 2.

Notes were synthesised using a version of the Karplus–Strong string synthesis procedure,^1^ which is based on a physical model of a plucked string (Karplus and Strong 1983). When played correctly, each note activates with an initial high intensity and decays roughly exponentially to total silence in approximately 1000 ms. An additional velocity mapping was included in this sonification procedure, whereby movements between corner zones which were excessively slow would prolong the length of the initial high-intensity impulse of the upcoming note while also reducing the ‘brightness’ of the sound. This feature was triggered when the maximum recorded velocity between two corners fell below a threshold of 0.17 m/s, after which the decaying current note could potentially be just audible beyond the onset of the next. In perception–action terms, this meant that when a participant did not stop at corner zones but rather continued in a slow, continuous fashion, consecutive notes would appear to blend together. Discrete movements (with peak velocity >0.17 m/s) between corners produced a short-duration initial impulse with a ‘bright’ quality, which meant that consecutive notes were perceptually discrete. No specific instructions were given to participants regarding how to move between corners in any of the three conditions.

The demo animation for the melodic sonification condition was sonified as if movements were performed with an acceptably high velocity, i.e., with no extra duration. During both the demo presentation and live movement sonification, tones produced by movement of the left hand were panned to the left channel of the headphones and vice versa.

In the rhythmic sonification condition, corner arrivals were sonified with bursts of white noise. Loudness of the burst was modulated by an envelope function which reduced loudness exponentially until silence at 350 ms after onset. The demo animation was sonified with the same sounds. As in the melodic sonification condition, left-hand-produced sound was panned to the left and vice versa.

**Fig. 3:**
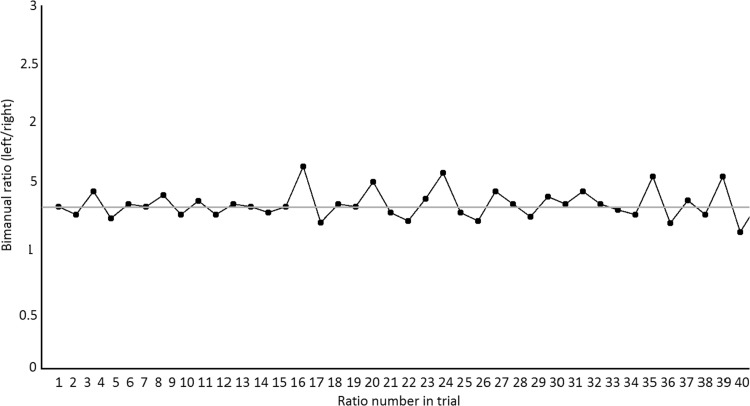
Post-trial feedback graph presented to participants following each trial in the practice stage. The *horizontal midline* corresponds to perfect performance. Axis labels were not visible to participants

Every trial in the practice stage was concluded with the presentation of a line graph of performance (see Fig. 3), showing raw ratio data for the previous trial relative to perfect performance. Throughout each trial, inter-corner intervals for the right hand were calculated and compared to the previous inter-corner interval on the left hand to produce a ratio. The ideal right-to-left ratio (3:4) was displayed on the graph as a green horizontal line across the centre of the screen. Participant-produced ratios were displayed as dots connected by a line.

### Procedure

Participants were each randomly allocated to one of the three experimental conditions (*N* = 20 in each). The experiment proceeded in seven stages for each participant.

### Task

Participants were instructed to move between corner zones at a regular rate on each hand, starting from the top, going anticlockwise. They were also told that a full rotation/cycle of both shapes should be completed within the same length of time, i.e., both index fingers should return to the top corner together. This requires movements of either hand to be independent of each other in terms of their temporal execution, but coupled in their relative timing (Summers et al. 1993).

### Stage 1: familiarisation

As in Dyer et al. (2016), the visual demo animation was played twice without sound prior to the practice phase. This animation showed the corners corresponding to the apparatus shapes lighting up in sequence, demonstrating the spatiotemporal requirements of the movement task. The top corners of both shapes lit up simultaneously. Corners on the left (triangle) then lit up once every 1000 ms, while corners on the right (diamond) lit up every 750 ms, both in an anticlockwise direction. A full cycle thus lasted 3 s exactly. Every play of the demo (including in the later practice phase) consisted of three cycles (totalling 9 s in length). For familiarisation, it was played twice (18 s total). Participants were then given time to attempt to produce the movements that they had observed in the demo without their performance being recorded (approximately 15 s). No sound was presented during familiarisation.

### Stage 2: pre-test

Participants in all conditions completed a single trial under control conditions to ensure equality of performance, on average, at the outset. This trial was performed while listening to pink noise through headphones during the demo and movement phases. The demo was played once (3 cycles, 9 s total), and then, participants were given 26 s in which to produce the movements of the task. During this time, they were required to produce continuous cycles on the shapes. No feedback (auditory or graphical) was provided on this trial.

### Stage 3: practice

The procedure for practice trials did not differ between groups except in terms of the auditory information available. Practice trials commenced with a play of the demo, followed immediately by a 26-s recorded movement phase and concluded with the presentation of the terminal feedback graph (Fig. 3). Fourteen practice trials were completed in total.

In the melodic and rhythmic sonification conditions, the demo and participant movements were sonified as described earlier, such that perfect performance by participants produced exactly the same sound heard during the demo. In the control condition, constant task-irrelevant pink noise was heard throughout the demo and movement phases.

### Stage 4: short-term retention (post-test)

Following a break of 5 min, a 26-s retention test was administered without a demo presentation or any form of feedback (neither sonification nor graph). During this time, participants were invited to perform the task to the best of their ability while listening to constant pink noise.

### Stage 5: 24-h retention

The following day, participants returned to the lab to repeat the retention test from the day before exactly.

### Stage 6: 24-h post-replay retention

Participants performed another retention test under the same conditions as previously described. However, prior to movement, participants in the two sonification conditions heard the sound produced by perfect performance of the task according to their condition (i.e., those in the melodic sonification condition heard the melodic demo, and so on). The sound of the demo was played twice, without any accompanying visuals (6 cycles, 18 s of sound total). Participants in the control condition did not hear nor see any task-related information prior to their test.

### Stage 7: transfer

Transfer of learning from a learned to an unlearned but similar task is generally taken as an indicator of robust learning (Soderstrom and Bjork 2015). A transfer test was conducted here to determine whether there might be differential transfer of learning between feedback conditions. The test involved the switching of the shapes. The triangle was placed on the right and the diamond on the left. The task goals were the same (4:3 bimanual rhythmic shape tracing), and only the apparatus was mirrored.

### Analyses

The main measure of performance in the current experiment is bimanual timing ratio error. For each individual trial, a series of ratios were calculated by comparing the inter-corner timing interval across hands. These raw ratios are the same as those presented to participants as terminal/post-trial feedback (see Fig. 3, for example). The absolute differences between each of these ratios and the required (4:3) ratio were averaged to produce a single score of average absolute ratio error for each trial. Analysis in this experiment focuses mainly on detecting potential benefits of sonification (either type) relative to the control condition.

ANOVA are employed to test for differences in performance between feedback conditions at relevant timepoints in the experiment. Rates of learning for each condition were examined using linear regression on learning curves produced from performance in the practice stage (trials 1–14).

Retention data were subjected to a confidence interval-based statistical test of non-inferiority. This allows comparison of the efficacy of a new intervention to the efficacy of an already established effective intervention (both relative to control). This procedure is appropriate for statistically verifying that the performance benefit seen at trial 14 in the melodic sonification condition (relative to control) is not lost when sonified feedback is withdrawn—and that there is no evidence of a guidance effect. This procedure is described in full in Walker and Nowacki (2011); see also Dyer et al. (2016). Briefly, if the 90% upper confidence interval (CI) of the difference in error scores between trial 14 and the later retention test falls within a predefined non-inferiority interval, then it can be statistically inferred (at the 0.05 level) that participant performance does not decline without feedback. In this case, the non-inferiority interval is set at 0.085, which is 0.5 times the difference in mean scores between the melodic sonification condition and control at trial 14 (Walker and Nowacki 2011).

Two participants in the control condition were unable to perform the transfer task sufficiently to allow measurement of performance; similarly, one participant in the rhythmic condition was unable to perform the task at pre-test. Therefore, these three trials were omitted from the analysis.

## Results

### Pre-test

Bimanual ratio error rates at pre-test were similar between experimental conditions: in the melodic condition, mean error rate was 0.39, SD = 0.16; in the rhythmic condition, mean = 0.42, SD = 0.16; and in the control condition, mean = 0.50, SD = 0.31. A one-way ANOVA on data from the pre-test revealed no significant effect of feedback condition on scores: *F*(2, 58) = 1.090, *p* = 0.344. From this, it can be inferred that performance did not differ between groups prior to practice.

### Practice trials 1–14

A mixed ANOVA across all acquisition data (trials 1–14, all participants) with feedback condition as a between-group factor and practice trial as a repeated measure factor revealed a significant main effect of trial: *F*(6.059, 260.525) = 7.867, *p* < 0.001, *η* ^2^ = 0.107 but not of group: *F*(2, 43) = 2.589, *p* = 0.087. A significant interaction effect of trial*group was found: *F*(12.117, 260.525) = 2.818, *p* = 0.001, *η* ^2^ = 0.116. Pairwise comparisons of group performance were performed on data from the final practice trial (14) to test for the effect of sonification on performance by the end of the practice stage, while sonification was still available (α was set at 0.016—Bonferroni correction for three comparisons). These indicated that participants in the melodic sonification condition performed significantly better than those in the rhythmic sonification condition: *p* = 0.004, Cohen’s *d* = 1.06; and the control condition: *p* = 0.001, Cohen’s *d* = 0.959. Performance did not differ significantly between the control and rhythmic sonification conditions: *p* = 0.593.

Rate of learning is a central concern here; therefore, linear regression with trial as a predictor was performed on each of the three learning curves comprising participant performance from practice trials 1–14. A significant model was found in both sonification conditions (in the melodic sonification condition: *F*(1, 266) = 55.760, *p* < 0.001 and in the rhythmic sonification condition: *F*(1, 271) = 23.541, *p* < 0.001), but not in the control condition: *F*(1, 276) = 0.840, *p* = 0.360. Standardised *β*-coefficients imply a faster rate of learning in the melodic sonification condition (*β* = −0.416, *t*(19) = −7.467, *p* < 0.001) than the rhythmic sonification condition (*β* = −0.283, *t*(19) = −4.849, *p* < 0.001) and the control condition (*β* = −0.055, *t*(19) = −0.916, *p* = 0.360). This indicates that while trial significantly predicted performance when sonification was available, there was not a similarly consistent change in performance over trials 1–14 in the control condition. Furthermore, learners in the rhythmic sonification condition did not achieve as great a reduction in error over the course of the practice phase as did those in the melodic sonification condition. There was little if any reduction in average ratio error over the course of 14 practice trials in the control condition.

### Retention testing

At the 5-min retention test (administered without feedback), the mean bimanual ratio error score in the melodic sonification condition was 0.17, SD = 0.11; in the rhythmic condition, mean = 0.33, SD = 0.21; and in the control condition, mean = 0.32, SD = 0.22. Change in mean scores between the initial pre-test and 5-min retention indicates some performance improvement in all experimental conditions.

**Fig. 4:**
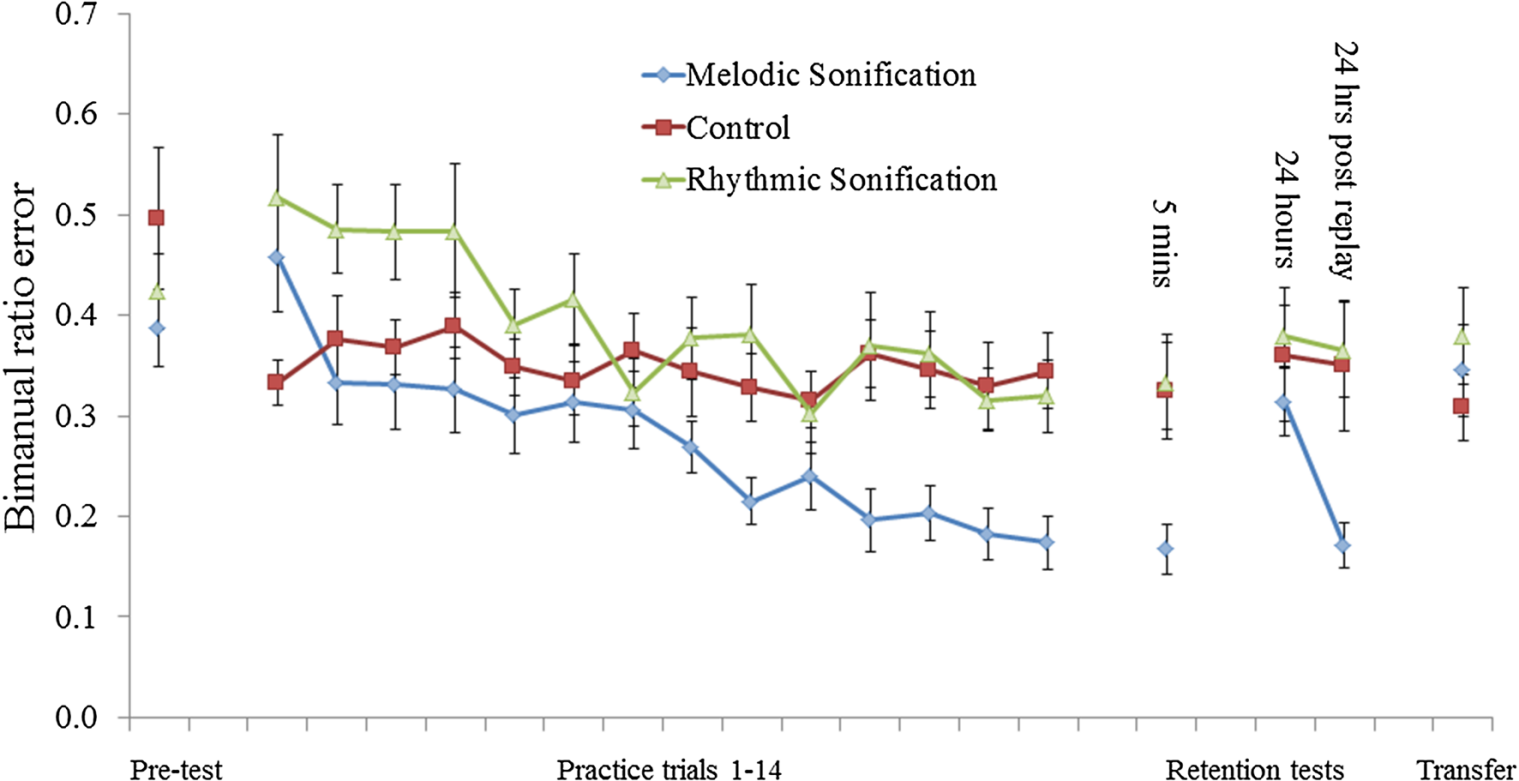
Rates of average absolute bimanual ratio error per condition in pre-test, practice, retention testing, and transfer testing for each of the three training condition groups. A score of 0 represents perfect performance. *Error bars* are standard error

The mean of the difference scores for the melodic sonification condition between trial 14 and the 5-min retention test was 0.01, with a 90% CI of [−0.01, 0.03]. The upper confidence interval is 0.03, which falls below the non-inferiority interval of 0.085. A *p* value is provided for this test by performing a one-sided, one sample *t* test on difference scores relative to the non-inferiority interval, 0.085: *t*(19) = −6.98, *p* < 0.001. It can, therefore, be inferred that the improved performance relative to control observed in the melodic sonification condition did not diminish without the presence of sound (see Fig. 4).

At the first 24-h retention test, ANOVA revealed no significant effect of feedback condition on error scores: *F*(2, 59) = 0.588, *p* = 0.558. The improved performance in the melodic sonification group was not evident after 24 h.

Following the sonic replay, a significant effect of feedback condition on error scores was again detected: *F*(2, 59) = 5.208, *p* = 0.008. Post-hoc *t* tests showed that this effect was driven by significantly lower error scores (*α* = 0.016) in the melodic sonification condition relative to control (*p* = 0.010) and rhythmic sonification (*p* = 0.006). Performance did not differ between rhythmic sonification and control (*p* = 0.830). A further test of non-inferiority relative to performance at trial 14 was performed on data from the melodic sonification condition following the replay. The mean of the difference scores between these two points was 0.002, with a 90 CI of [−0.03, 0.03]. The upper CI falls within the 0.085 threshold for non-inferiority (*t*(19) = −5.319, *p* < 0.001); therefore, it can be inferred that performance in the melodic sonification condition was no worse after 24 h and a sonic replay than at the final practice trial.

To test for a potential practice effect of repeated performance on retention test scores, data from 24-h retention (pre- and post-replay) from the control condition were subjected to a paired samples *t* test. No change in performance between these two performance tests was evident. The mean score at the first of the two tests was 0.362 (SD = 0.291) and at the second was 0.350 (SD = 0.284); *t*(19) = 0.553, *p* = 0.587.

### Transfer testing

ANOVA revealed no significant effect of feedback condition on performance in the transfer test: *F*(2,57) = 0.648, *p* = 0.527.

## Discussion

In this experiment, sonification of movement which employed a melodic mapping was more effective for motor skill learning than a similarly-structured sonification which consisted only of basic temporal (rhythmic) information. By the end of practice, average bimanual ratio error was significantly lower in the melodic sonification condition than both the rhythmic condition and the control condition. The rhythmic sonification, which was expected to improve performance by more clearly specifying the timing of required and produced participant movements, did not improve performance relative to control. This result indicates that the improved performance observed in the sonification condition in Dyer et al. (2016) was not only due to action–sound coupling, but an action–sound coupling which produced a meaningful melodic pattern. Separate components of this experiment’s results will here be discussed in turn.

That rhythmic sonification of movement did not produce an improvement in performance at all relative to control is somewhat surprising. From the sensorimotor timing and motor control literature, it is clear that actions performed in the presence of a sonic metronome, or intrinsically, sounding actions can be performed with greater temporal accuracy than similar actions performed in silence (Kennel et al. 2015; Repp and Penel 2002). Good performance on this task (which shares some fundamental characteristics with classic polyrhythmic coordination tasks, see Summers et al. 1993) is at least partly dependent on the fine temporal control which is afforded by action–sound coupling. The timing structure of the auditory information provided in this rhythmic sonification condition was essentially the same as that provided in the melodic sonification condition and the sonification condition in Dyer et al. (2016). Sound events were coupled directly to movement events and the resulting auditory information was not transformed or abstracted from the basic underlying kinematics of the task—which should have made it directly useful for the coordination of action (Chiou and Chang 2016). However, it is important to note that the present task is more complex than tasks typically used in the bimanual tapping paradigm (Kennedy et al. 2013; Klapp et al. 1998). Most of the research which informs the above assumption (that attaching any sound to movements will improve rhythmic bimanual coordination performance) comes from uni- or bimanual finger tapping on static force plates, positioned directly beneath the hands. No comparable research into the effect of purely rhythmic sonification on polyrhythmic bimanual coordination in more complex tasks yet exists (to the author’s knowledge). The requirements of the current task include perceptually conflicting features such as movements in different directions, amplitudes, using non-homologous muscles and at different times—all of which are known to increase coordination difficulty (Shea et al. 2016; Swinnen and Gooijers 2015). Thus, the level of complexity of the current task is greater than that of tasks typically used in auditory–motor timing and coordination research. The surprising finding of a lack of performance improvement in the rhythmic sonification condition relative to control may be explained by the above-mentioned differences between the ‘simpler’ kinds of tasks used in contemporary research and the more complex task used in current study (for a detailed analysis of why we might not expect to see results generalise from ‘simple’ tasks to ‘complex’ tasks, see Wulf and Shea 2002).

In this task, the benefit of melody can be conceptualised in terms of the extra, useful information that it provides in addition to inter-movement interval durations. With the melodic sonification mapping, the auditory feedback provided varies on an additional auditory dimension (musical pitch) compared to the rhythmic mapping. The rhythmic mapping provides primarily timing information, whereas the melodic mapping additionally provides positional information to a learner in motion. Attaching specific notes to individual corner targets clearly specifies current positioning and the order in which subsequent movements must be performed. Mistakes in the ordering of movements are reflected in very salient mistakes in the unfolding melody produced during the cycle. On the following cycle, this can be corrected. The use of a melody in sonified feedback might allow the use of an anticipatory strategy in motor control: the correct next movement is known to a learner who is familiar with the system—it is the move which will continue/resolve the ongoing melodic pattern. Ordering mistakes are reflected in the main measure of bimanual ratio error, as movements out-of-order are movements which come too soon or too late, and thereby affect the bimanual timing ratio. Conversely, with the rhythmic sonification, it is possible to produce a rhythm very close to that presented in the demo, while still making ordering mistakes in the execution of the task (aside from the lateral stereo panning, which would reveal the error, however, this is likely much less salient than mistakes in a melody). It was predicted that the informational benefit for melody in this task would manifest as a faster rate of learning in the melodic sonification condition, which it did. β-coefficients derived from regression analysis of the learning curves show that learning was faster in the presence of melodic information than rhythmic information. It is possible that, given more time, participants in the rhythmic sonification condition could have learned to use the spatial, stereo-panned information to better control the ordering of their movements; however, with the necessary specifying information being so subtle, it is unclear how long such improvement would take.

In the 5-min retention test, with all feedback removed, task performance was unchanged. The most interesting result from the first retention test is that the enhanced performance seen in the melodic sonification condition was not dependent on the presence of live feedback. Participants were no worse at the task without sonification. This is a replication of the main finding from Dyer et al. (2016), showing the absence of an early-retention guidance effect. Similar results have been reported by Ronsse et al. (2011) and van Vugt and Tillmann (2015) who directly sonified sequential actions and observed maintenance of good performance after the removal of sound.

On the 24-h retention test, the improved performance in the melodic sonification condition had disappeared; no effect of condition on performance was detected at this point. This was expected, and lines up with an identical finding in Dyer et al. (2016). In the previous study, participants frequently reported that they were unable to remember the melody, and blamed that for the decline in their performance. It was these reports and the notion of perception and action as a holistic process which inspired the attempt in the current study to prolong retention with the use of a sonic replay. The behavioural and neural crossover between perception of sound and action production in musical skill is very well-established (Lahav et al. 2007; Lotze et al. 2003; Taylor and Witt 2015). In piano learning, retention of a learned sequence of notes can be enhanced by motionless listening to the sound of correct performance (Lahav et al. 2013). A major strength of sonification as a vehicle for the delivery of augmented feedback information is that it can very easily transform non-musical tasks (with more abstract performance goals than the production of music) into musical tasks with features akin to traditional musicianship.

Following only 18 s of listening (two plays of the demo), differences between feedback conditions became evident again. Participants in the melodic sonification condition performed the task with lower rates of error than both control and the rhythmic sonification condition. In fact, a statistical test of non-inferiority showed that performance in the melodic sonification condition at this point was not any worse than that it had been at the very end of the practice stage on the day before. This indicates that, despite the poorer performance shown on the initial 24-h retention test, the motor skill did in fact remain in the repertoire. In this experiment, melody represented the key to recovering good motor performance from model observation after it had waned.

Beyond this experiment, the broader application of this finding is that novel motor skills can be trained with sonification and that good performance can be refreshed by listening to its ideal sound. However, no corresponding benefit of listening to a sonic replay was evident in the rhythmic sonification condition. Again, this is likely due to the information contained in sound, and the degree to which it specifies the movements of the task. The sound of the melody specifies the ordering of the movements of the task, but crucially, only for those participants who are skilled enough to perceive that information and its relevance (Carello et al. 2005; Steenson and Rodger 2015). A demonstration of task performance through feedback which is more abstract, or does not as precisely specify the interaction would likely not be as effective for refreshing motor performance. The sound of the rhythmic sonification demo in the current experiment may not clearly enough specify the ordering of the movements sonified for the sound alone to be useful in the same way as the melody—the sounds used are perhaps too generic and not easily distinguishable as caused by specific limbs in specific motion.

This finding has been implicitly recognised in other work which has used sonification. For example, Ronsse et al. (2011) opted for two-tone sonification (successful performance of their task produced four evenly-spaced tones, in what sounded like a galloping rhythm) rather than one, which would have been as generic as the rhythmic sonification in the current experiment. Four evenly-spaced tones with identical sound would have been much less meaningful and more difficult to use to coordinate a difficult bimanual skill. Melodic information as simple as variation in pitch between two beeps (for two hand orientations) may be sufficient to clearly specify task requirements, as it is in Ronsse et al. (2011); however, more complex motor skill sonification may require a correspondingly elaborate melody. The present study cannot rule this possibility out, and therefore, it remains an open question in need of further investigation.

Performance in the transfer test did not differ between experimental conditions. Despite the fact that participants in the melodic sonification condition were able to improve their performance on the main task to a level equivalent to the previous day, this did not affect performance on a mirrored version of the task.

There are a number of limitations to the present study. First, because movement sonification as augmented feedback was compared to a no-sound control group, it does not allow us to determine whether learning in this task would have been enhanced if participants moved with the model demo sound during training trials. That is, it is not clear whether the sound needs to be self-generated to lead to enhanced learning, or whether synchronising movements to the model melody would be sufficient. Second, in the melodic sonification condition, the velocity of participants’ movements could alter the characteristics of the offsets of the triggered notes if they fell below a certain threshold. Although this does not provide any relative timing information, and so would be unlikely to enhance learning of the task, it is possible that the more complex mapping between actions and sound may have influenced performance in this group. Finally, further research is necessary to determine the extent to which the advantages of melodic sonification for complex motor learning found in this study can apply to different complex skills, particularly skills with greater real-world application.

## Conclusion

The current study presents the benefits of melodic sonification for learning of a novel motor skill. The use of melody in the practice phase allowed participants in that condition to reach significantly lower error scores than control, and a sonification which used only rhythmic information. It has been argued that the main mechanism driving this effect is the extra information contained in melody and its ability to specify the ordering of the task, whereas purely rhythmic information does not allow for this. The secondary finding is that after performance has declined on a musical sonification-trained task, performance of the pattern can be recovered by listening to the sound of a perfectly-performed demonstration. Re-exposure to an augmented feedback-enhanced learning environment is not necessary if the skill is reconceptualised as a musical task. These findings have important implications for understanding the role of augmented perceptual information in skill learning, as well as its application to real-world training or rehabilitation scenarios.

## Footnotes

1 Patch available online at: http://bit.ly/2hnBGFv.

## References

[CR1] Anderson DI, Magill RA, Sekiya H, Ryan G (2005). Support for an explanation of the guidance effect in motor skill learning. J Motor Behav.

[CR2] Bangert M, Peschel T, Schlaug G (2006). Shared networks for auditory and motor processing in professional pianists: evidence from fMRI conjunction. NeuroImage.

[CR3] Carello C, Wagman J, Turvey M, Fisher Anderson B, Anderson JD (2005). Acoustic specification of object properties. Moving image theory: ecological considerations.

[CR4] Cesari P, Camponogara I, Papetti S, Rocchesso D, Fontana F (2014). Might as well jump: sound affects muscle activation in skateboarding. PLoS One.

[CR5] Chen JL, Rae C, Watkins KE (2012). Learning to play a melody: an fMRI study examining the formation of auditory-motor associations. NeuroImage.

[CR6] Chiou SC, Chang EC (2016). Bimanual coordination learning with different augmented feedback modalities and information types. PLoS One.

[CR7] Dyer J, Stapleton P, Rodger M (2015). Sonification as concurrent augmented feedback for motor skill learning and the importance of mapping design. Open Psychol J.

[CR8] Dyer J, Stapleton P, Rodger MWM (2016). Transposing musical skill: sonification of movement as concurrent augmented feedback enhances learning in a bimanual task. Psychol Res.

[CR9] Effenberg AO (2005). Movement sonification: effects on perception and action. IEEE Multimed.

[CR10] Franz EA, Zelaznik HN, Swinnen SP, Walter CB (2001). Spatial conceptual influences on the coordination of bimanual actions: when a dual task becomes a single task. J Motor Behav.

[CR11] Gibson EJ (1969). Principles of perceptual learning and development.

[CR12] Gibson EJ (1988). Exploratory behavior in the development of perceiving, acting, and the acquiring of knowledge. Annu Rev Psychol.

[CR13] Karplus K, Strong A (1983). Digital synthesis of and plucked-string timbres. Comput Music J.

[CR14] Kennedy DM, Boyle JB, Shea CH (2013). The role of auditory and visual models in the production of bimanual tapping patterns. Exp Brain Res.

[CR15] Kennel C, Streese L, Pizzera A, Justen C, Hohmann T, Raab M (2015). Auditory reafferences: the influence of real-time feedback on movement control. Front Psychol.

[CR16] Klapp ST, Nelson JM, Jagacinski RJ (1998). Can people tap concurrent bimanual rhythms independently?. J Motor Behav.

[CR17] Kovacs AJ, Buchanan JJ, Shea CH (2009). Bimanual 1:1 with 90° continuous relative phase: difficult or easy!. Exp Brain Res.

[CR18] Kovacs AJ, Buchanan JJ, Shea CH (2010). Impossible is nothing: 5:3 and 4:3 multi-frequency bimanual coordination. Exp Brain Res.

[CR19] Lahav A, Saltzman E, Schlaug G (2007). Action representation of sound: audiomotor recognition network while listening to newly acquired actions. J Neurosci.

[CR20] Lahav A, Katz T, Chess R, Saltzman E (2013). Improved motor sequence retention by motionless listening. Psychol Res-Psychol Forsch.

[CR21] Lotze M, Scheler G, Tan H-R, Braun C, Birbaumer N (2003). The musician’s brain: functional imaging of amateurs and professionals during performance and imagery. NeuroImage.

[CR22] Maslovat D, Brunke KM, Chua R, Franks IM (2009). Feedback effects on learning a novel bimanual coordination pattern: support for the guidance hypothesis. J Motor Behav.

[CR23] Mechsner F, Kerzel D, Knoblich È, Prinz W (2001). Perceptual basis of bimanual coordination. Nature.

[CR24] Oldfield R (1971). The assessment and analysis of handedness: the Edinburgh inventory. Neuropsychologia.

[CR25] Repp BH, Penel A (2002). Auditory dominance in temporal processing: new evidence from synchronization with simultaneous visual and auditory sequences. J Exp Psychol Human.

[CR26] Ronsse R, Puttemans V, Coxon JP, Goble DJ, Wagemans J, Wenderoth N, Swinnen SP (2011). Motor learning with augmented feedback: modality-dependent behavioral and neural consequences. Cereb Cortex.

[CR27] Salmoni AW, Schmidt RA, Walter CB (1984). Knowledge of results and motor learning: a review and critical reappraisal. Psychol Bull.

[CR51] Shea CH, Buchanan JJ, Kennedy DM (2016). Perception and action influences on discrete and reciprocal bimanual coordination. Psychon Bull Rev.

[CR28] Sigrist R, Rauter G, Riener R, Wolf P (2013). Augmented visual, auditory, haptic, and multimodal feedback in motor learning: a review. Psychon Bull Rev.

[CR29] Sigrist R, Rauter G, Riener R, Wolf P (2013). Terminal feedback outperforms concurrent visual, auditory, and haptic feedback in learning a complex rowing-type task. J Motor Behav.

[CR30] Soderstrom NC, Bjork RA (2015). Learning versus performance: an integrative review. Perspect Psychol Sci.

[CR31] Steenson C, Rodger M (2015). Bringing sounds into use: thinking of sounds as materials and a sketch of auditory affordances. Open Psychol J.

[CR32] Summers JJ, Rosenbaum DA, Burns BD, Ford SK (1993). Production of polyrhythms. J Exp Psychol Human.

[CR33] Swinnen SP, Gooijers J, Toga AW (2015). Bimanual coordination. Brain mapping: an encyclopedic reference.

[CR34] Taylor JET, Witt JK (2015). Listening to music primes space: pianists, but not novices, simulate heard actions. Psychol Res-Psychol Forsch.

[CR35] Todorov E, Shadmehr R, Bizzi E (1997). Augmented feedback presented in a virtual environment accelerates learning of a difficult motor task. J Motor Behav.

[CR36] van Vugt FT, Tillmann B (2015). Auditory feedback in error-based learning of motor regularity. Brain Res.

[CR50] Vander Linden DW, Cauraugh JH, Greene TA (1993) The effect of frequency of kinetic feedback on learning an isometric force production task in nondisabled subjects. Phys Ther 73(2):79–87 10.1093/ptj/73.2.79 8421721

[CR37] Vinken PM, Kröger D, Fehse U, Schmitz G, Brock H, Effenberg AO (2013). Auditory coding of human movement kinematics. Multisensory Res.

[CR38] Walker E, Nowacki AS (2011). Understanding equivalence and noninferiority testing. J Gen Intern Med.

[CR39] Wilson AD, Collins DR, Bingham GP (2005). Perceptual coupling in rhythmic movement coordination: stable perception leads to stable action. Exp Brain Res.

[CR40] Wilson AD, Snapp-Childs W, Coats R, Bingham GP (2010). Learning a coordinated rhythmic movement with task-appropriate coordination feedback. Exp Brain Res.

[CR41] Wulf G, Shea CH (2002). Principles derived from the study of simple skills do not generalize to complex skill learning. Psychon Bull Rev.

